# Development of a Transformation System for *Nitratireductor* sp.

**DOI:** 10.1007/s10126-023-10198-4

**Published:** 2023-02-03

**Authors:** Hiroto Maeda, Yuto Hirata, Hirokazu Takahashi, Kenshi Watanabe, Tsunehiro Aki, Yoshiko Okamura

**Affiliations:** Graduate School of Integrated Sciences for Life, Hiroshima University, 1-3-1 Kagamiyama, Higashi-Hiroshima, Hiroshima, 739-8530 Japan

**Keywords:** Butenoic acid, Electroporation, *Nitratireductor*, Self-cloning, Transformation

## Abstract

*Nitratireductor* sp. OM-1 can accumulate butenoic acid, which is a short-chain unsaturated carboxylic acid utilized for chemical products. So far, we have predicted the thioesterase gene, *te*, as a candidate gene for butenoic acid biosynthesis, based on comparative transcriptome analysis. To confirm the function of *te*, the gene transfer system in *Nitratireductor* sp. OM-1 was required. Thus, in this study, we used electroporation as a transformation system and pRK415, a broad host range plasmid, and optimized the conditions. As a result, a maximum transformation efficiency of 7.9 × 10^4^ colonies/µg DNA was obtained at 22.5 kV/cm. Moreover, an expression vector, pRK415-*te*, was constructed by insertion of *te*, which was successfully transferred into strain OM-1, using electroporation. The recombinant OM-1 strain produced butenoic acid at 26.7 mg/g of dried cell weight, which was a 254% increase compared to transformants harboring an empty vector. This is the first report of a gene transfer system for *Nitratireductor* sp., which showed that the *te* gene was responsible for butenoic acid production.

**Supplementary Information:**

The online version contains supplementary material available at 10.1007/s10126-023-10198-4.

## Introduction

Butenoic acid (crotonic acid) is an important C4 short-chain unsaturated carboxylic acid. It has been widely used for industrial applications, including pharmaceuticals, resins, agrochemicals, and cosmetics. Currently, butenoic acid is synthesized by oxidation of crotonic aldehyde, which is produced by condensation of acetaldehyde obtained from petroleum resources. However, when naphtha is used as the starting material, the yield is as low as 30% (Mamat et al. 2014). Moreover, non-renewable processes are currently a major drawback. Butenoic acid synthesis by fermentation would solve this problem. From biological sources, butenoic acid can be obtained by pyrolysis of poly(3-hydroxybutyrate) (PHB) (Mamat et al. 2014). However, there are very few reports of direct biosynthesis of butenoic acid in microorganisms. The first report by Dellomonaco et al. (2011), expected to be the most ideal approach, focused on reversing the β-oxidation pathway. They established an engineered strain RB02, which was modified for reverse operation of the pathway of wild-type *Escherichia coli* MG1655 (genotype; fadR atoC(c) crp* ∆arcA ∆pta ∆adhE ∆frdA) and then introduced *tesA* encoding thioesterase I and *yqeF*, encoding a predicted acyltransferase. As the result of extensive genetic engineering, the mutant strain successfully accumulated *trans*-butenoic acid at 200 mg/L (Dellomonaco et al. 2011). On the other hand, Liu et al. (2015) used the *Bacteroides thetaiotaomicron* thioesterase gene (*bTE*), which could hydrolyze four carbon acyl-ACPs (Jing et al. 2011) to improve butenoic acid production. They introduced *bTE* into *fadD*-deleted mutant of *E. coli*, inhibited enoyl-ACP reductase (FabI) by triclosan and achieved accumulation of butenoic acid at 161.4 mg/L (Liu et al. 2015). In the latest study, *Yarrowia lipolytica* was used as a heterologous expression host, with introduced crotonase and 3-hydroxybutyryl-CoA dehydrogenase genes from *Clostridium beijerinckii* and *bTE*, which resulted in 62.3 ± 4.2 mg/L of butenoic acid (Wang et al. 2019). Furthermore, they included acetyl-CoA acetyltransferase and pyruvate dehydrogenase by additional heterologous expression and achieved 220 mg/L of butenoic acid (Wang et al. 2019).

We have reported that our isolate, *Nitratireductor* sp. OM-1, produced significant amounts of butenoic acid during nitrogen-depleted conditions (Okamura et al. 2016, 2019). This was the first report of a non-engineered bacterium that accumulated butenoic acid. Moreover, ester compounds consisting of butenoic acid were also highly accumulated at 50% of total lipid (Okamura et al. 2016). Notably, the OM-1 strain prefers short-chain fatty acids or volatile fatty acids (VFAs) over glucose. It was hypothesized that it converted VFAs into butenoic acid and its ester. Whole genome analysis revealed that the *te* gene, showing homology with *bTE*, had an enhanced expression level which increased 4.07-fold during nitrogen-depleted conditions when compared with nitrogen-supplemented conditions (Okamura et al. 2019). This result strongly suggested that *te* was responsible for butenoic acid synthesis in OM-1. Moreover, based on the productivity of butenoic acid in the OM-1 strain, *te* is expected to have higher enzyme activity than *bTE*. We also have attempted to introduce *te* from the OM-1 strain into *E. coli*; however, the growth of the recombinant strain containing *te* was strongly inhibited (unpublished). A transformation system in *Nitratireductor* sp. OM-1 is therefore required for self-cloning of *te*, because it has a high tolerance toward butenoic acid.

In the future, development of a transformation system is essential for the identification of the unique OM-1 lipid synthesis pathway. In this study, we therefore aimed to develop a vector introduction system for OM-1 and to identify the function of *te*.

## Materials and Methods

### Bacterial Strains, Media, Vectors, and Culture Conditions

Bacterial strains and vectors used in this study are listed in Table 1. *Nitratireductor* sp. OM-1 was grown in ATCC medium #1409 according to a previous study (Okamura et al. 2016). Carbon sources were properly changed from 1 g/L sodium acetate to 5 g/L glycerol, 5 g/L valeric acid, or 6.06 g/L acetic acid and 1.43 g/L propionic acid, depending on the purposes of experiments, as indicated in each figure legend. For lipid accumulation, the concentration of yeast extract in the pre-culture was changed from 0.1 g/L yeast extract to that of 5 g/L, and NH_4_Cl was omitted from the medium in the main culture. *E. coli* strain DH5α was used for cloning and maintaining in LB medium. A broad host range vector for gram-negative bacteria, pRK415, and its derivative vector, pRK415-*te*, were introduced into both strains and maintained in each medium supplemented with 12.5 µg/mL tetracycline (Nacalai Tesque, Kyoto, Japan). For the preparation of competent *Nitratireductor* sp. OM-1 cells, glycerol was used as a carbon source. All culturing of the OM-1 strain involved incubation at 28 °C with shaking at 100 rpm, with an initial inoculum ratio of 1:100.

**Table 1:** Bacterial strains and plasmids used in this study

Strains or plasmids	Description	Reference or source
Strains		
*Nitratireductor* sp. OM-1	Wild-type	Okamura et al. (2016)
*E. coli* DH5α	*deo*R, *sup*E44, *hsd*R17(r_k_^−^, m_k_^+^), *pho*A, *rec*A1, *end*A1, *gyr*A96, *thi-1*, *rel*A1, D(*lac*ZYA-*arg*F) U169, f80d*lac*ZDM15, F^−^, l^−^	TOYOBO, Osaka, Japan
Plasmids		
pRK415	Broad host range vector for gram-negative bacteria; Tet^r^	Life Science Market
pRK415-*te*	PCR product encoding the *te* gene with putative promoter was cloned into *Eco*RI/*Pst*I-digested pRK415; Tet^r^	This study

### Construction of an Expression Vector

The *te* gene and the predicted promoter region were amplified with the primers listed in Table 2. The BPROM function of Softberry, an online software, was used for promoter prediction. The PCR mixture included 10 µM of each primer, 10^5^ copies of genome DNA from *Nitratireductor* sp. OM-1, and the KOD One® PCR Master Mix-Blue (Toyobo, Osaka, Japan). The thermal cycling conditions were as follows: predenaturation at 95 °C for 2 min, followed by 35 cycles of denaturation at 98 °C for 10 s, annealing and extension at 68 °C for 5 s, with an additional extension at 72 °C for 4 min. The thermal cycler was a T100 Thermal Cycler (Bio-Rad, Hercules, CA, USA). The resulting PCR product was ligated into the *Eco*RI/*Pst*I site in the multi-cloning site of pRK415 using a TaKaRa DNA Ligation Kit LONG (Takara Bio, Shiga, Japan) and cloned in *E. coli* DH5α. The resulting clones of transformants were tested by colony PCR using an M13 primer set. The PCR products were then resolved using an agarose gel electrophoresis [1%, Tris–acetate EDTA (TAE) buffer] and viewed using a Blue Light Transilluminator (LEDB-SBOXH; OptoCode, Tokyo, Japan). The positive clones were cultivated for plasmid extraction, and the plasmid was extracted using a Pure Yield™Plasmid Midiprep System (Promega, Madison, WI, USA). The plasmid was sent for sequencing to Eurofins Genomics (Tokyo, Japan).

**Table 2:** Oligonucleotide primers used in this study

Primer	Sequence (5′-3′)	Reference or source
P_*te*_-*te*-HA^ab^	F-GAGAGCTGCAGACACATTCTCAAGTTCGATCACGG R-GAGAGAATTCTTA*GGCATAGTCGGGCACGTCATAGGGATA*GGCGTCGTCTCCCTGATGCGCTTCGAT	This study
M13	F-TGTAAAACGACGGCCAGT R-CAGGAAACAGCTATGACC	Yanisch-Perron et al. (1985)

^a^Restriction enzyme recognition sites are underlined

^b^Hemagglutinin tag (HA-tag) sequences are indicated in italics

### Electroporation

Electroporation against the OM-1 strain was performed using a Gene Pulser, ECM®630 (BTX®, Holliston, MA, USA) at a capacitance of 25 µF and a resistance of 200 Ω using 0.1 cm cuvettes. Harvested cells of the OM-1 strain were washed with 10 mM HEPES buffer containing 272 mM sucrose (pH 7.0) (Okamura et al. 2003) and resuspended in the same buffer at a 200 × concentration ratio. A total of 50 µL of cell suspension was aliquoted and used as electrocompetent cells. An appropriate amount of plasmid DNA was added to an aliquot, and 20 µL of the resulting suspension was transferred to a cuvette. The cells were subjected to single-pulse electroporation, then transferred into 1 mL of #1409 medium with 20 mM Mg^2+^ and incubated at 28 °C for 10 h with shaking at 280 rpm. A 100 µL culture was then plated on 1% agar in #1409 medium containing 12.5 µg/mL tetracycline and incubated at 28 °C for 3 days. Transformation efficiency was calculated as the number of colony formation units (CFUs)/µg-DNA.

### Lipid Analysis

The OM-1 cells were grown in 80 mL of culture medium for 5 days and then harvested and lyophilized using an FDU-1200 lyophilizer (Eyela, Tokyo, Japan). Chloroform:methanol [2:1 (v/v)] was added to the lyophilized cells, and the chloroform layer was collected after vortexing. These operations were repeated four times, and a double distilled water was added to the extracted solution and vortexed. After centrifugation, the chloroform layer was again collected. The solvent was completely evaporated under nitrogen on a heat block at 60 °C and then total lipids were obtained and weighed to calculate lipid content and productivity. Because butenoic acid showed peak tailing, high-performance gas chromatograph-time-of-flight mass spectrometry (GC-TOF–MS) (JMS-T100 GCV; Jeol, Tokyo, Japan) was used to identify the components in the total lipid extract based on the molecular mass and the set of fragment masses (Okamura et al. 2019). The column, a DB-5ht column (Agilent Technologies, Santa Clara, CA, USA) was used. The temperature increases involved 100 °C (held for 2 min) to 340 °C at 10 °C/min. The injection method was the split injection method (split ratio: 1:10), and the solvent flush method was used to prevent discoloration. The inlet temperature was 300 °C, the injection volume was 1 µL, the carrier gas was helium (99.999% purity), and the column flow rate was set at 1.2 mL/min. Mass spectrometry was performed by electron ionization (70 eV) with a mass range of m/z 29–800. MassCenter, version 2.6.4 (Jeol), and the Mass Spectral Library (NIST11, National Institute of Standard and Technology) were used for static analysis. In addition, GC using the internal standard method was performed to compare the weight of butenoic acid in total lipids from the self-cloning and wild-type strains. As an internal standard lipid, 75 µg arachidic acid (Nacalai Tesque, Kyoto, Japan) was added to 200 µL of 10 mg/mL total lipid. Energy-saving capillary gas chromatography (GC-2025; Shimadzu, Kyoto, Japan) was used for analysis, and the column was same as used in GC–MS. The temperature increase conditions were 40 to 340 °C (held for 7 min) at 10 °C/min. The injection method was the split injection method (split ratio: 1:5). The injection volume was 1 µL, the carrier gas was nitrogen, and the column flow rate was set at 1.62 mL/min. GCSolution software, version 2.4 (Shimadzu) was used for static analysis. The weight of butenoic acid was calculated from the obtained peak areas of arachidic acid and each component.

### Other Measurements

Bacterial growth was evaluated by measuring the OD_660_ of the culture using an ANA-18A + Model Colorimeter (Koden, Tokyo, Japan). Statistical analysis was determined by the Student’s *t*-test (*P* < 0.05).

## Results and Discussions

### Confirmation of Resistances Against Tetracycline and Pulsed Electric Field in the OM-1 Strain

To date, there has been no report of transformation systems for *Nitratireductor* sp. At first, before using a broad host range vector for gram-negative bacteria, pRK415, the growth of the OM-1 strain supplemented with tetracycline was examined to determine its resistance against selection pressure. OD_660_ of the OM-1 culture without tetracycline reached at 2.0 for 36 h; however, the growth of the OM-1 strain supplemented with tetracycline was not observed for 60 h. Thus, tetracycline could be used as a selection pressure for self-cloning using pRK415.

Next, we examined the strength of a pulsed electric field, from which *Nitratireductor* sp. was able to recover.

**Fig. 1:**
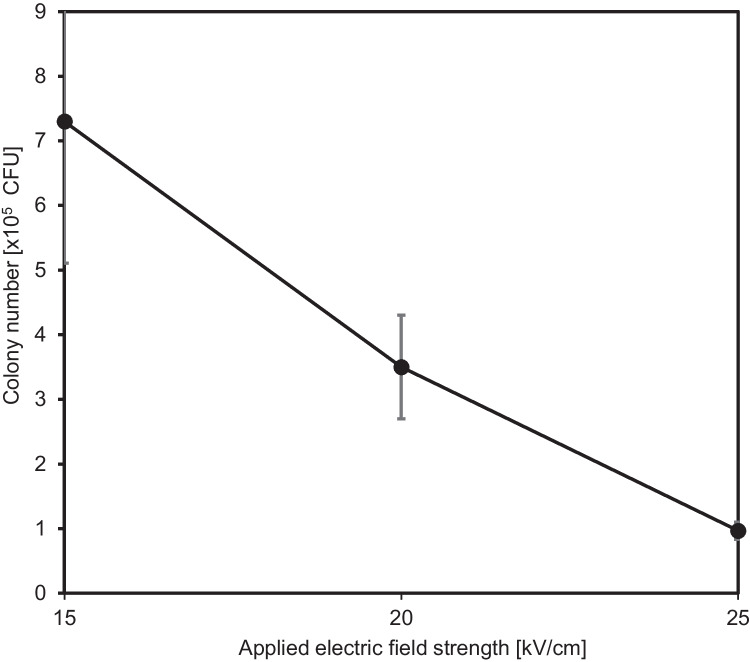
The resistance of electropulses of *Nitratireductor* sp. OM-1 (*N* = 3). OM-1 was grown on the agar plate supplemented with glycerol as the sole carbon source and formed colonies without recovery culture after being treated with an electric field strength from 15 to 25 kV/cm

Initially, the electric field strength was applied to wild-type *Nitratireductor* sp. at 15, 20, or 25 kV/cm, and the electrocharged cells were spread on the agar plate without incubation for membrane repair. The results showed that the OM-1 strain was resistant to a pulse less than 25 kV/cm (Fig. 1), suggesting that electroporation could be used to introduce DNA into the *Nitratireductor* sp.

### Confirmation of pRK415 Maintenance in the OM-1 Strain

We subsequently introduced pRK415 into the OM-1 strain using electroporation. Before optimization the conditions, electroporation was performed using 200 ng of pRK415 and an electric field strength at 20 kV/cm. After recovery culture for 10 h, the cells were grown on a tetracycline-containing agar plate. To confirm the maintenance of plasmids, several colonies were picked and cultivated in liquid medium, and then plasmids were extracted. The restriction fragments with a total length of 10.69 kb from pRK415 were confirmed by electrophoresis.

### Consideration of Electroporation Conditions

Transformation efficiency is the most important factor in genetic recombination. To improve efficiency, we considered the DNA amount, electric field strength, and duration of the recovery culture (Calvin and Hanawalt 1988).

We first focused on optimizing the amount of plasmid DNA. The relationship between the amount of added DNA and CFU is summarized in Table 3. Colony formation was not observed with 0.5 nor 1 ng of plasmid, suggesting that the cell competency was low. However, 16 colonies were observed when using 100 ng of DNA (4.0 × 10^3^ CFU/µg-DNA). Therefore, in this study, more than 100 ng DNA was used for the subsequent experiments.

**Table 3:** Relationship between additional amount of DNA and colony number (*N* = 3); the increase of DNA amount; colony formation units were also increased

**Added DNA amount (ng)**	**Formed colony number (*N*** **= 3**)
0.5	0 ± 0.000
1	0 ± 0.000
5	1 ± 0.000
10	1 ± 0.001
100	16 ± 0.004

**Fig. 2:**
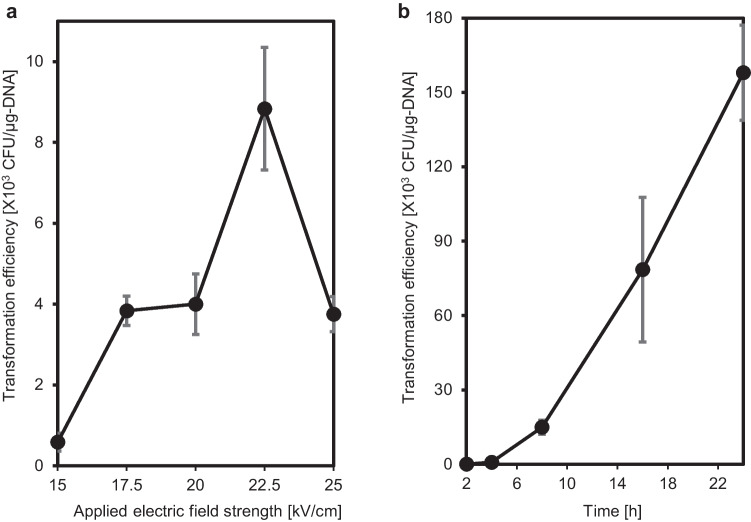
**a** Relationship between applied electric field strength and transformation efficiency (*N* = 3). **b** Relationship between duration of the recovery culture and transformation efficiency (*N* = 3)

Second, we focused on the applied electric field strength as another important factor for transformation efficiency, because it directly affects the size of the cell membrane pore, and optimized strength depends on the types of microorganisms (Manas and Pagan 2005). The effects of applied electric field strength on transformation efficiency are shown in Fig. 2a. Transformation efficiency increased with increasing electric field strength from 15 to 22.5 kV/cm. This was attributed to the stronger electric field, which enlarged the size of membrane pores, favoring plasmid uptake. In contrast, treatment with 25 kV/cm showed a decrease in transformation efficiency. This might be because the higher electric field strength resulted in irreparable damage to the cell membranes, resulting in cell death. Therefore, we concluded that 22.5 kV/cm, which resulted in the maximum transformation efficiency of 8.8 × 10^3^ CFU/µg-DNA, was the optimal condition treatment.

Third, we examined culture recovery time, which promotes cell membrane repair prior to exposure to the selection pressure of antibiotics. The effects of recovery time on transformation efficiency at 22.5 kV/cm are shown in Fig. 2b. An increase in transformation efficiency with increasing recovery time showed that 2 ~ 4 h was not enough time to repair the cell membranes, as the cells did not adequately survive exposure to tetracycline. There was an increase in transformation efficiency at 8, 16, and 24 h, indicating that duration of recovery culture should be longer to obtain more colonies; however, cell division started after 20 h. Therefore, based on the sufficient transformation efficiency of 7.9 × 10^4^ CFU/µg-DNA and the convenience of time management, 16 h was used in the experiments. By using these parameters, the efficiency of transformation was improved and was used in the following studies.

### Expression of Exogenous Thioesterase by Self-cloning

**Fig. 3:**
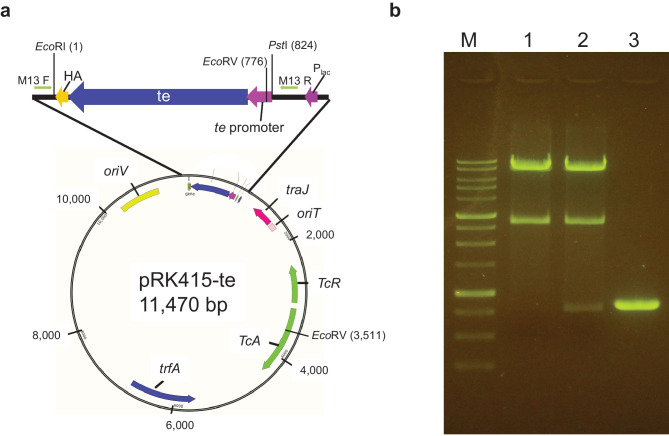
**a** Physical map of the expression vector pRK415-*te*. The PCR fragment was inserted at *Eco*RI and *Pst*I sites. **b** Confirmation of the expression vector, pRK415-*te*. The pRK415-*te* and empty vector were digested by *Eco*RI, *Eco*RV, and *Pst*I. M: 1 kb DNA ladder, 1: pRK415, 2: pRK415-*te*, 3: PCR product of the insert

The butenoic acid synthesis pathway shares part of the fatty acid synthesis pathway where (2E)-butenoyl-ACP is converted into butenoic acid, which is catalyzed by thioesterase (Liu et al. 2015). The thioesterase gene *te* with an upstream region, including a putative promotor, was amplified and ligated with pRK415 at multiple cloning site, with the resulting plasmid designated as pRK415-*te* (Fig. 3a)*.* Construction of the expression vector was confirmed by electrophoresis (Fig. 3b). The sequence of the insert of pRK415-*te* was determined, and sequence accuracy was confirmed.

**Fig. 4:**
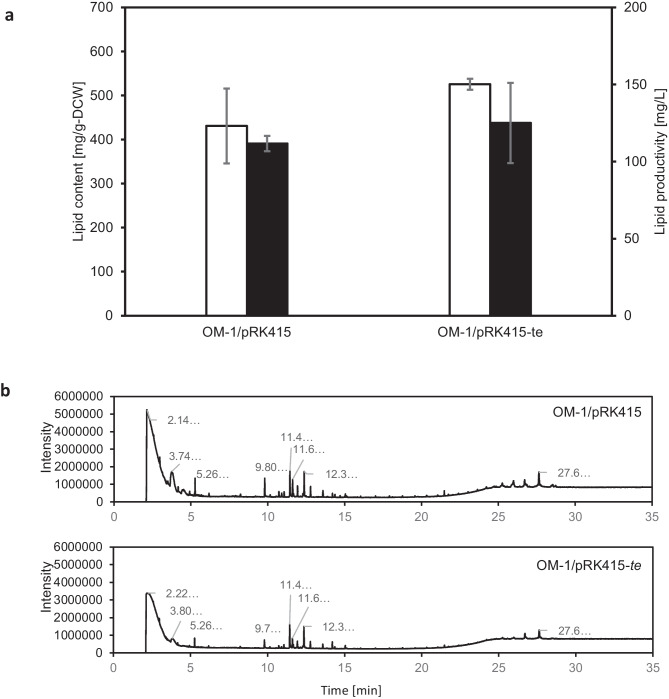
**a** Lipid content and productivity of OM-1/pRK415 and OM-1/pRK415-*te* during lipid accumulation conditions (*N* = 6). White bar: lipid content (mg/g-DCW), black bar: lipid productivity (mg/L). *P* value of lipid content and productivity by *t*-test was 0.3314 and 0.6414, respectively. Both recombinants were grown with 5 g/L valeric acid in the pre-culture and with 6.06 g/L acetic acid and 1.43 g/L propionic acid in the main culture. **b** Lipid composition measured by gas chromatography-mass spectrometry. From top to bottom: OM-1/pRK415 and OM-1/pRK415-*te* during lipid accumulation conditions. The butenoic acid peak appeared at around RT 2.13 (determined by GC-TOF–MS)

The *te* gene is upregulated fourfold during nitrogen-depleted conditions (Okamura et al. 2019). Thus, both endogenous and exogenous *te* was induced in a nitrogen-depleted medium. Total lipid was extracted from both recombinants, and lipid production per volume culture medium and lipid content per dry cell weight (DCW) were calculated using the lipid weight, culture volume, and DCW, respectively. The lipid content per DCW and lipid productivity showed insignificant differences in both strains when using the *t*-test (Fig. 4a). We, therefore, determined the amount of butenoic acid using the internal standard method. Butenoic acid contents per dried cell weight in OM-1-harboring pRK415 and pRK415-*te* were 10.5 mg/g-DCW and 26.7 mg/g-DCW, respectively, indicating a 254% increase when compared with empty vector transformant (Table 4). This result supports our hypothesis that (2E)-butenoyl-ACP, an intermediate in fatty acid synthesis, is used to produce butenoic acid in the presence of exogenous thioesterase, resulting in increased butenoic acid accumulation. This amount is not high; however, strain OM-1 can also produce PHB, which consists of 85% of total lipid (in preparation). The recombinant strain containing pRK415-te produced 526 mg/g-DCW of total lipid, meaning 447 mg/g-DCW would be PHB content, 79 mg/g-DCW would be the separable content according to GC, and 34% of total separable lipids corresponded to the butenoic acid content. Moreover, the lipid composition analyzed by GC-TOF–MS showed no difference between the recombinants (Fig. 4b). Together, the results suggested that the amount of butenoic acid might be controlled, and that excess butenoic acid might be converted into polymers.

**Table 4:** Comparison of butenoic acid production between OM-1/prK415-*te* and OM-1/prK415 (*N* = 6)

**Strains**	**Butenoic acid content**	
	**In total lipid (%)**	**In dry cell weight (DCW) (mg/g-DCW)**
OM-1/pRK415	2.46 ± 0.72	10.5 ± 2.9
OM-1/pRK415-*te*	5.06 ± 0.35	26.7 ± 4.6

*P* value of “In dry cell weight” by *t*-test was 0.0408

With the rapid progress of genetic recombination and genome editing in recent years, the method for gene transfer is useful. However, applying the gene introduction system of *E. coli* laboratory strains to bacteria isolated from the environment has not always been successful. The gene transfer system for *Nitratireductor* sp. was successfully developed in this study and will be helpful to identify the functions of unknown genes.

## Conclusions

In this study, we have successfully developed a vector introduction system for *Nitratireductor* sp. OM-1 using pRK415. Through considerations of transformation conditions, the maximum transformation efficiency of 7.9 × 10^4^ CFU/µg-DNA was obtained. These results established a research base to maximize the industrial value of OM-1, which is thought to possess unique pathways such as butenoic acid and its ester synthesis pathways. This method might be commonly available for other strains of *Nitratireductor* sp.

Through this system, we established a self-cloning recombinant strain to overexpress thioesterase and improve butenoic acid production. Strain OM-1 harboring pRK415-*te* produced a 254% increase in butenoic acid compared with control recombinant strains.

### Supplementary Information

Below is the link to the electronic supplementary material.

Supplementary file1 (DOCX 375 KB)

## Data Availability

The data that supports the findings of this study are available in the supplementary material of this article.
