# Supplementary figures and images for: Development of a Transformation System for Nitratireductor sp
Source: Mar Biotechnol (NY). 2023 Feb 3;25(5):644–51. doi: 10.1007/s10126-023-10198-4 (PMC10665240; doi:10.1007/s10126-023-10198-4)

M 1　 2 3


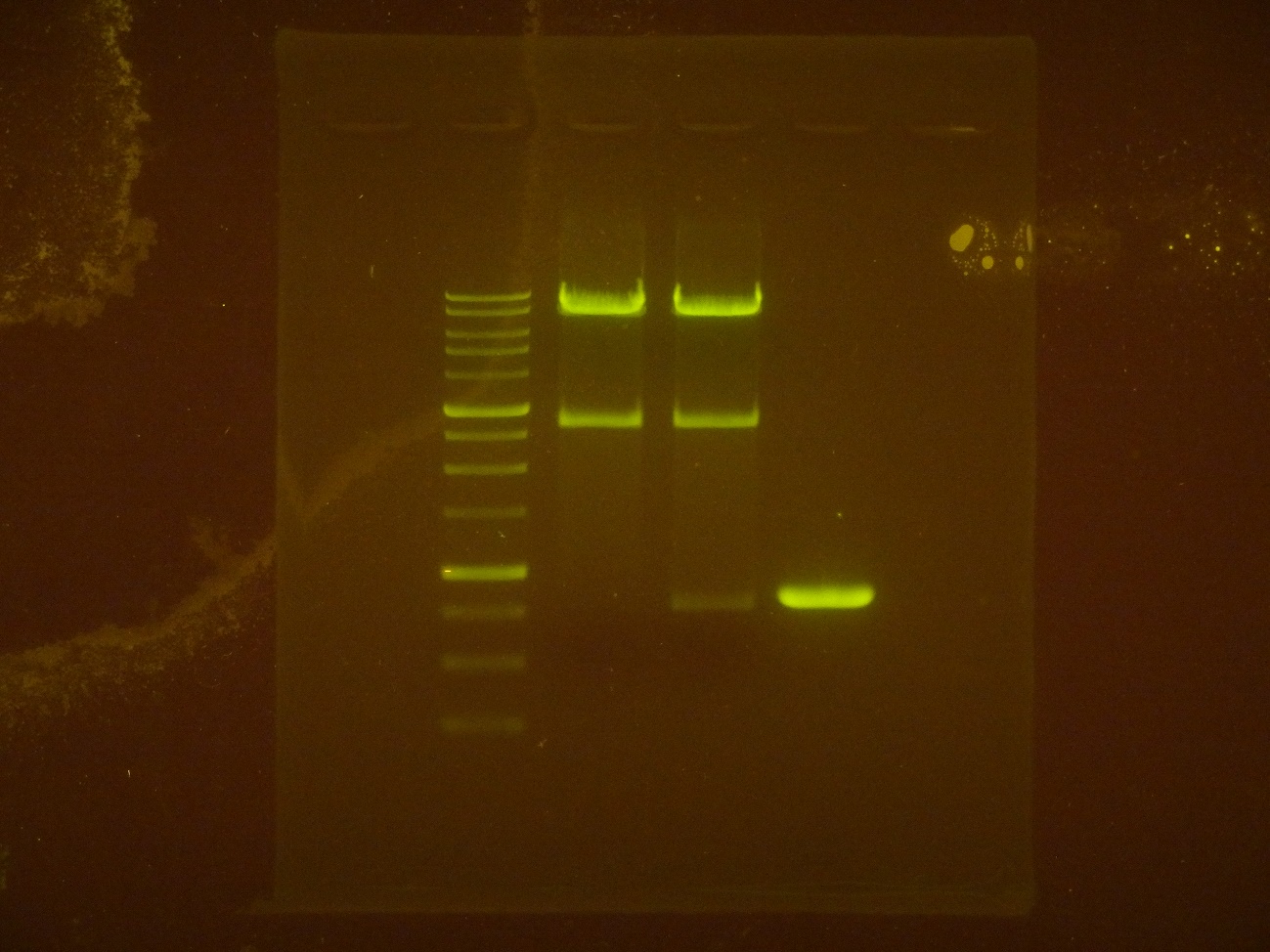


**Supplementary Figure 1. Non-trimmed image of agarose gel electrophoresis (same as Figure 3b).**

Supplement: Supplementary file 1 — Supplementary file1 (DOCX 375 KB) [file 10126_2023_10198_MOESM1_ESM.docx]
